# Real-Time Characterization of Clonal Fate Decisions in Complex Leukemia Samples by Fluorescent Genetic Barcoding

**DOI:** 10.3390/cells11244045

**Published:** 2022-12-14

**Authors:** Tobias Maetzig, Anna Lieske, Nicole Dörpmund, Michael Rothe, Marc-Jens Kleppa, Violetta Dziadek, Jacob Jalil Hassan, Julia Dahlke, Dorit Borchert, Axel Schambach

**Affiliations:** 1 Institute of Experimental Hematology, Hannover Medical School, 30625 Hannover, Germany; 2 Department of Pediatric Hematology and Oncology, Hannover Medical School, 30625 Hannover, Germany; 3 Division of Hematology/Oncology, Boston Children’s Hospital, Harvard Medical School, Boston, MA 02115, USA

**Keywords:** flow cytometry, clonal tracking, fluorescent genetic barcoding, acute myeloid leukemia

## Abstract

Clonal heterogeneity in acute myeloid leukemia (AML) forms the basis for treatment failure and relapse. Attempts to decipher clonal evolution and clonal competition primarily depend on deep sequencing approaches. However, this prevents the experimental confirmation of the identified disease-relevant traits on the same cell material. Here, we describe the development and application of a complex fluorescent genetic barcoding (cFGB) lentiviral vector system for the labeling and subsequent multiplex tracking of up to 48 viable AML clones by flow cytometry. This approach allowed the visualization of longitudinal changes in the in vitro growth behavior of multiplexed color-coded AML clones for up to 137 days. Functional studies of flow cytometry-enriched clones documented their stably inherited increase in competitiveness, despite the absence of growth-promoting mutations in exome sequencing data. Transplantation of aliquots of a color-coded AML cell mix into mice revealed the initial engraftment of similar clones and their subsequent differential distribution in the animals over time. Targeted RNA-sequencing of paired pre-malignant and de novo expanded clones linked gene sets associated with Myc-targets, embryonic stem cells, and RAS signaling to the foundation of clonal expansion. These results demonstrate the potency of cFGB-mediated clonal tracking for the deconvolution of verifiable driver-mechanisms underlying clonal selection in leukemia.

## 1. Introduction

Acute myeloid leukemia (AML) originates from the acquisition of differentiation blocking and proliferation-promoting mutations in the hematopoietic stem and progenitor (HSPC) compartment [1]. After the emergence of the first transformed clone, Darwinian selection and branching clonal evolution continue to drive the production of a heterogeneous pool of leukemic stem cells (LSC) with defined mutational and transcriptional signatures [2]. The resulting clonal heterogeneity challenges therapeutic interventions because of the associated risk for pre-existing or emerging therapy-resistant clones, with relapsed and unresponsive patients, in particular, having a poor prognosis [3,4,5,6]. Although patient prognosis and long-term cures would conceivably benefit from a better understanding of clonal evolution, the underlying selection mechanisms during unperturbed leukemogenesis and therapeutic challenge remain obscure. This may, in part, relate to the difficulties of recapitulating clonal evolution under defined experimental conditions and the sequencing of bulk populations for the assessment of mutational loads and associated transcriptional properties [7,8,9]. Recently, single-cell sequencing enabled a more precise assessment of the mutual exclusivity and co-occurrence of mutations, the derivation of clonal pedigrees at various stages of disease progression, and the transcriptional uniqueness of AML clones [2,10,11,12]. Regardless, the functional characterization of dedicated mutants from sequencing studies remains challenging, due to a lack of techniques that would facilitate the isolation of viable cells of interest.

In an attempt to enable the tracking and re-isolation of viable cell clones, the Fehse laboratory developed an optical barcoding (OBC) approach that relies on the combinatorial integration of multiple fluorescent protein-encoding lentiviral vectors into bulk cell populations, followed by single-cell sorting of clones with defined fluorescent properties and tracking their fate by flow cytometry after cell mixing [13]. This approach provided insights into the clonal heterogeneity of glioblastoma cells as a function of immune status [14]. In parallel, our group advanced lentiviral fluorescent genetic barcoding (FGB) vector systems that follow the one vector per color code concept and produce 6 to 14 flow cytometry-compatible labels suitable for the characterization of hematopoietic cells [15,16].

In an aggressive *Hoxa9* and *Meis1* (H9M)-dependent murine AML model that recapitulates human disease, FGB-assisted multiplex assays readily allowed for the assessment of the competitive behavior of polyclonal H9M AML samples during leukemogenesis, drug exposure and miRNA challenge [15]. Surprisingly, most in vivo multiplex assays produced a single dominant color-coded population of unknown clonal complexity within 4–8 weeks. Given the aggressive nature of H9M AML, its dependency on the sole overexpression of *Hoxa9* and *Meis1*, and its anticipated genetic stability, the expansion of dedicated color codes suggests the acquisition of a limited number of H9M collaborating genetic aberrations or transcriptional adaptations with putative prognostic value for human AML [17,18]. However, the low complexity of the previously utilized FGB vector system does not support the monitoring of mutant formation at clonal resolution for empirically linking cellular behavior to alleged leukemogenic (transcriptional and genetic) features. 

To enable these studies, we here describe the development of a complex FGB (cFGB) vector system for real-time multiplex tracking of up to 48 viable cell clones. We illustrate the applicability and reliability of our cFGB labeling approach by flow cytometry-guided parallel tracking of up to 48 H9M AML clones in vitro and up to 24 clones in vivo based on the maximum number of available color-coding vectors and the highest achievable specificity, respectively. Since previous DNA-barcoding-mediated tracking experiments of AML typically detected less than 50 dominant clones at end-stage disease, our color-coding complexity appears sufficient to label a representative number of clones for functional studies [19]. This was demonstrated through multiplex competition assays by the identification of growth-enhanced mutants, their re-isolation from cell mixtures, and linkage of clonal identity to genetic and transcriptional profiles.

## 2. Materials and Methods

### 2.1. Generation and Cultivation of H9M Cell Lines

Lineage negative (Lin^−^) bone marrow cells were purified from congenic C57BL/6-Ly5.1 mice with the MojoSort Mouse Hematopoietic Progenitor Cell Isolation Kit (Biolegend, San Diego, CA, USA), according to the manufacturer’s instructions. Lin^−^ cells were subsequently pre-stimulated for 2 days in basal medium (Dulbecco’s modified Eagle’s medium (DMEM; Biochrom, Berlin, Germany) with 15% (*v*/*v*) fetal bovine serum (FBS) (PanBiotech, Aidenach, Germany), 100 U/mL penicillin and 100 µg/mL streptomycin and 1 mM sodium pyruvate (PanBiotech)), supplemented with 6 ng/mL murine interleukin 3 (mIL-3), 10 ng/mL human interleukin 6 (hIL-6) and 20 ng/mL murine stem cell factor (mSCF) (=36SF medium; all cytokines from PeproTech GmbH, Hamburg, Germany). Afterwards, 5 × 10^4^ cells were seeded into 96 well round bottom plates with 100 µL 36SF medium supplemented with 4 µg/mL protamine sulfate for overnight transduction with a VSVg-pseudotyped gammaretroviral vector (pRSF91-Meis1-2A-Hoxa9.i2.Puro.LVpre) expressing murine cDNAs for *Meis1*, *Hoxa9* and the puromycin-resistance *(pac)* gene [20]. After 2 days, cells were selected with 1 µg/mL puromycin for 1 week, after which the cells were grown in 36SF medium. For cytokine challenge experiments, cells were grown in basal medium supplemented with 50 ng/mL human granulocyte colony stimulating factor (hG-CSF) (PeproTech). For colony-forming cell assays (CFA), 1 × 10^3^ cells were seeded into 1 mL of methylcellulose supplemented with myeloid cytokines (MC#3434, STEMCELL Technologies, Vancouver, BC, Canada). After 1 week, colonies were rinsed off the plates, washed and stained for flow cytometric analysis.

### 2.2. Nucleofection

Nucleofections of 1 × 10^6^ H9M cells for inactivation of murine *Dhfr* (dihydrofolate reductase) were performed according to the protocol by Hendel et al. for CRISPR-Cas9 RNP delivery to CD34^+^ hematopoietic stem and progenitor cells from the Integrated DNA Technologies (IDT; Coralville, IA, USA) website (https://eu.idtdna.com/pages, accessed on 20 September 2019) using the Amaxa 4D-Nucleofector (Lonza, Basel, Switzerland) and protocol DN100. Cas9, tracrRNA and crRNA against murine *Dhfr* (crRNA-DHFR.1AD gcaagaacggagacctaccc(TGG)) were purchased from IDT. After nucleofection, cells were resuspended in 36SF medium with and without 1:100 HT-supplement (Gibco, Waltham, MA, USA) for cultivation and expansion. For analysis of gene editing rates, cell pellets were harvested and subjected to direct PCR amplification of the *Dhfr* locus using primers DHFR-AD1_FW 5′-TAGCTGCACAAATAGGATGCGCG-3′ and DHFR-AD1_RV 5′-TATGCTCAGGCTCCATTCAGCG-3′ and the Phire Tissue Direct PCR Master Mix (Thermo Scientific, Waltham, MA, USA), according to the “Animal tissues—Dilution & Storage” protocol. PCR products were loaded on an agarose gel, excised, purified and subjected to Sanger sequencing using primer DHFR-AD1_RV. Gene editing rates in relation to a non-edited PCR product were assessed using TIDE [21].

### 2.3. Lentiviral Vector Cloning and Production

Vectors were cloned according to standard procedures. CAARs consist of a signaling peptide derived from the low-affinity nerve growth factor receptor (LNGFR), followed by 2-fold repeats of hemagglutinin (HA) and cMyc tags surrounding the antibody-binding domain of Thy1.1 (dThy1.1). These elements were fused to a signaling peptide-deprived truncated epidermal growth factor receptor (EGFRt) [22], which serves as membrane anchor. All tags are separated by flexible 2x(glycin-glycin-glycin-serin) [(G3S)2] linkers, and non-antibody binding permutations carry amino acid substitutions that keep the length of each fragment constant. The cDNA sequence of the modified humanized monomeric Azami green (hmAG3) was generated from hmAG1 by introducing the GFP-derived 3′ BsrGI restriction site and stop codon, as well as destruction of an internal BbsI restriction site by silent nucleotide exchange [23]. Likewise, mCherrEY was generated by removing the internal NcoI and BbsI restriction sites from the parental mCherry cDNA by silent nucleotide exchange. These modifications were required to render fluorescent protein sequences compatible with BbsI-mediated Golden Gate Assembly, as well as to ease cloning via NcoI and BsrGI restriction sites. Cloning details are available on request.

Vector production was performed in 293T or 293TRAP cells. The latter cells were generated by stably transfecting 293T cells with a linearized plasmid encoding for a human codon-optimized *TRAP* (hTRAPco; tryptophan RNA-binding attenuation protein) cDNA and subsequent puromycin selection [24]. Binding of the TRAP protein to the vector-encoded trap binding site (tbs) reduces transgene expression in producer cells and increases vector titers. For vector production, 7–9 µg lentiviral FGB-(D) vector, 9 µg pcDNA3.g/p.4xCTE, 6 µg pRSV-Rev, and 1.5 µg pMD2.G (VSVg) plasmid were used for calcium phosphate mediated transfection of 10-cm cell culture dishes. Supernatants were harvested after 36 h and optionally after 60 h for ultracentrifugation or PEG-mediated precipitation [25]. Aliquots were stored at −80 °C until use.

### 2.4. Lentiviral Transductions

5 × 10^4^ H9M or H9M^−/−^ cells were seeded into 96 well round bottom plates in 100 µL 36SF medium supplemented with 4 µg/mL protamine sulfate and 1 mg/mL Poloxamer synperonic F108 (Sigma-Aldrich, St. Louis, MO, USA). After the addition of viral supernatants, the cells were placed into a 95% humidified incubator with 5% CO_2_ at 37 °C. For transduction of H9M^−/−^ cells, the transduction medium was additionally supplemented with 1:100 HT-reagent (Gibco, Waltham, MA, USA). Cells were washed 24 h after transduction and subsequently expanded until flow cytometric analysis, and/or initiation of tracking experiments, for which the cells were typically split into a 1:10 ratio every 2 to 3 days.

### 2.5. Flow Cytometric Analysis

Flow cytometric analyses were performed on a 4 laser (V-B-Y-R), 13 fluorescent channel CytoFLEX S (Beckman Coulter, Brea, CA, USA). Therefore, cells were washed, optionally stained for surface markers, and resuspended in FACS buffer (PBS, 2% (*v*/*v*) FBS and 4 mM EDTA) supplemented with 0.2 µg/mL 4′,6-diamidino-2-phenylindole (DAPI). For analyses, cells were first gated by SSC-A vs. FSC-A, prior to single cell discrimination via FSC-H vs. FSC-A, and FSC-A vs. DAPI for live/dead cell discrimination. Stainings were performed in 50 to 100 µL FACS buffer using antibodies cMyc-biotin (1:100, clone SH1-26E7.1.3, Miltenyi Biotech, Bergisch Gladbach, Germany), SAV-Brilliant Violet 605 (1:100, Biolegend, London, UK), Thy1.1-PECy7 (1:2500, clone OX-7, Biolegend), HA-PE (1:100, clone GG8-1F3.3, Miltenyi Biotech), and EGFR-APC (1:200, clone AY13, Biolegend).

For F-MTX assay, we adapted the original protocol by Santiago et al. by incubating equal numbers of cells in fresh 36SF medium supplemented with 1:100 HT reagent and 3 µM Fluorescein Methotrexate (Biotium, Fremont, CA, USA) for 2 h at 37 °C [26]. The medium was replaced with twice the amount of fresh 36SF-HT medium, and further incubated for 30 min. at 37 °C. Afterwards, the cells were washed with PBS and resuspended in FACS-buffer incl. 0.2 µg/mL DAPI for flow cytometric assessment of fluorescein signals in comparison to a wild-type control sample.

### 2.6. DNA Barcode Amplification and Sequencing

Genomic DNA from minor and dominant clones was purified using the QIAamp DNA Blood Mini Kit (QIAGEN, Hilden, Germany). 100 ng of genomic DNA of each sample was subsequently amplified with sample-specific indexing primers (pFGB-BCi5_FW and pFGB-BCi7_RV (10 µM each)) during three rounds of amplification (see Table S3 for primer sequences). PCR products >150 bp were subsequently enriched by size exclusion using the QIAGEN GeneRead Size Selection Kit, according to the manufacturer’s instructions. 1 µL purified DNA was used for nested PCR using universal primers pFGB-BC_outer_FW and pFGB-BC_outer_RV (10 µM each), and 30 cycles of amplification. All PCRs were performed with 2x Phusion Green Hot Start II High-Fidelity PCR Master Mix (Thermo Scientific). BC-specific PCR products (376 bp) were visualized by gel electrophoresis, excised and purified using QIAquick Gel Extraction Kit (QIAGEN). Sequencing reads were first filtered according to sample-specific indices prior to counting sequencing reads per BC.

### 2.7. Integration Site Analysis

Determination of the lentiviral integration sites in the mouse genome (mm9) used the INSPIIRED platform [27,28]. We used 1.6 µg genomic DNA as input material for shearing. Detailed descriptions of the specific preparation steps, PCR conditions, purifications, quality controls, and analytical procedures were described previously [29].

### 2.8. Exome Sequencing and Mutation Calling

1 μg genomic DNA per sample was used as input material for the DNA sample preparation. Sequencing libraries were generated using Agilent SureSelectXT Mouse All Exon kit (Agilent Technologies, Santa Clara, CA, USA), according to the manufacturer’s instructions. Sequencing was performed on an Illumina platform, followed by bioinformatics analyses. Exome sample preparation, sequencing, and data analysis were performed by Novogene (Cambridge, UK) according to established pipelines.

### 2.9. RNA-Sequencing and Raw Data Processing

Splenocytes from 24xFGB-D transplanted mice were thawed and directly sorted for defined color codes within the cKit^+^CD11b^dim^ gate into RLT buffer for subsequent purification with the RNeasy Micro Kit (QIAGEN). RNA was extracted from 6 × 10^3^–1 × 10^4^ cells per color-coded population, and 0.4–0.7 ng of total RNA was subsequently used for library preparation with the ‘SMARTer Stranded Total RNA-Seq Kit v3—Pico Input Mammalian—96 Rxns’ (#634487; Takara Bio USA, Inc., San Jose, CA, USA), according to conditions recommended in user manual #120720. Sequencing was performed on an Illumina NextSeq 550 sequencer using a High Output Flowcell for single reads (20024906; Illumina, San Diego, CA, USA). BCL files were converted to FASTQ files using bcl2fastq Conversion Software version v2.20.0.422 (Illumina), and raw data processing was conducted by use of nfcore/rnaseq (version 1.4.2). The genome reference and annotation data were taken from GENCODE.org (Mus musculus; GRCm38.p6; release M25). Normalization and differential expression analysis was performed with DESeq2 (Galaxy Tool Version 2.11.40.2) with default settings, except for “Output normalized counts table”, “Turn off outliers replacement”, “Turn off outliers filtering”, and “Turn off independent filtering”, all of which were set to “True”. Gene set enrichment analyses and heatmaps for differentially expressed genes were performed in GenePattern [30]. Raw and processed expression data have been deposited in the Gene Expression Omnibus under GEO: GSE198371.

### 2.10. Animal Experiments

For transplantation, each lethally irradiated (9 Gy) C57Bl6/J recipient was injected with a cell mix consisting of 1 × 10^5^ cells of each of the 24 color-coded H9M clones and 2 × 10^5^ radio-protective helper BM cells. The mice were monitored regularly and taken out of the experiment for organ harvest and analysis upon signs of terminal leukemia manifestation, according to approved protocols. All mice were maintained in a pathogen-free environment in the animal facility of Hannover Medical School.

## 3. Results

### 3.1. Design Considerations for a Complex Lentiviral Fluorescent Genetic Barcoding Vector System

**Figure 1:**
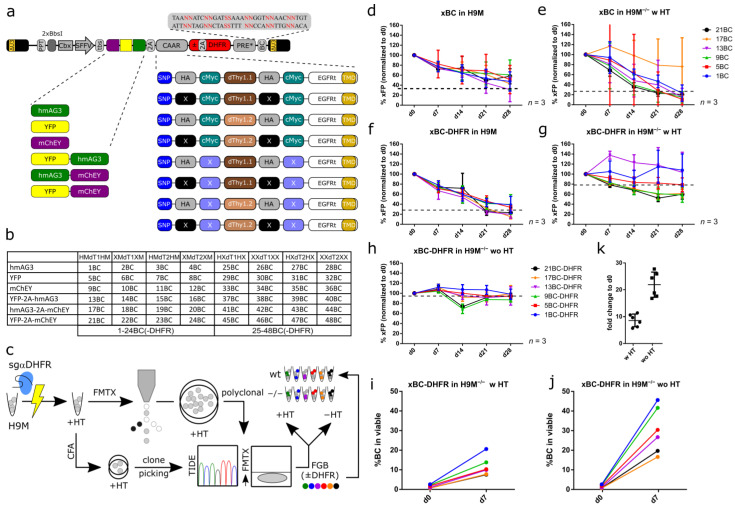
*DHFR* coexpressing FGB vectors are stably maintained in H9M^−/−^ cells. (**a**) Schematic representation of the 48xFGB(-D) lentiviral vector library. The color-coding cassette is driven from a compound promoter (CSF) consisting of the silencing resistant Cbx3 element fused to the spleen focus forming virus (SFFV) promoter. Color codes are generated through the expression of humanized monomeric Azami green (hmAG3), yellow fluorescent protein (YFP), and mCherrEY (mChEY) fluorescent protein. In addition, a chimeric antigen array (CAAR) is anchored to the cell surface via a truncated epidermal growth factor receptor (EGFRt) fused to hemagglutinin (HA) and cMyc tags, as well as the antibody binding domain of Thy1.1 (dThy1.1). Permutations (X/dThy1.2) of each module prevent antibody binding. The fluorescent marker—CAAR cassette is optionally followed by a dihydrofolate reductase (*DHFR*) cDNA, as well as a barcode (BC) between the post transcriptional element (PRE*) and the 3′ long terminal repeat (LTR). (**b**) Combinations of fluorescent marker, CAAR and BC of the 48xFGB(-D) lentiviral vector library. (**c**) Experimental design strategy. H9M *Dhfr* knockout cells (H9M^−/−^) were generated by RNP-mediated nucleofection of crisprRNA:tracrRNA complexed with Cas9. Cells were subsequently maintained in hypoxanthine and thymidine (HT)-supplemented medium prior to the derivation of pure knockout lines through CFA or sorting for fluorescein-labeled methotrexate negative cells. Resulting cultures were subsequently subjected to *Dhfr* locus amplification and evaluation of knockout rates by TIDE analysis. Samples with a validated knockout were subsequently transduced with FGB-(±DHFR) vectors. (**d**,**e**) FGB vector expression in (**d**) H9M and (**e**) H9M^−/−^ cells over time. (**f**–**h**) FGB-D vector expression in (**f**) H9M cells, as well as in H9M^−/−^ cells in the (**g**) presence and (**h**) absence of HT-supplementation over time. (**i**,**j**) Gene marking of FGB-D vectors in H9M^−/−^ cells in the (**i**) presence and (**j**) absence of HT-supplementation. The d0 values are shared between both analyses. (**k**) Fold change of gene marking rates between d0 and d7 based on data depicted in (**i**,**j**). Gene marking rates in (**d**–**h**) over time were normalized to the initial gene marking rate at the beginning of the assays (d0). PPT: polypurine tract; BbsI: restriction enzyme recognition site; tbs: trap binding sequence; 2A: 2A ribosomal skipping site; CAAR: chimeric antigen array; DHFR: dihydrofolate reductase; PRE*: post-transcriptional regulatory element; BC: barcode; SNP: signal peptide; TMD: transmembrane domain.

We envisioned the construction of a complex color-coding library based on the coexpression of fluorescent proteins and permutations of a chimeric array of surface marker tags. We designed this chimeric antigen array (CAAR) with a truncated epidermal growth factor receptor (EGFRt) that serves as membrane anchor and displays a tandem array of hemagglutinin (HA) and cMyc tags, as well as the antibody binding domain of Thy1.1 (= dThy1.1) [22]. For each element, a non-antibody binding variant (X for HA and cMyc or dThy1.2) was also constructed, resulting in eight different CAARs through combinatorial arrangement of antibody binding and non-binding permutations. The production of the full complexity of 48 different color codes for flow cytometric detection and longitudinal tracking required the coexpression of these CAARs with six fluorescent marker combinations consisting of monomeric Azami Green (hmAG3), yellow fluorescent protein (YFP), mCherrEY, YFP-2A-hmAG3, hmAG3-2A-mCherrEY, and YFP-2A-mCherrEY, similar to our previous studies (Figure 1a) [20,31]. Furthermore, each color-coded vector carries a unique DNA barcode (BC). The BC numbering will be used for the unequivocal identification of color-coded cells throughout the manuscript (Figure 1b and Table S1). 

For clonal tracking, reliable cell marking is of utmost importance, but preliminary experiments suggested that these 48xFGB vectors were not stably maintained over time. Therefore, we attempted to stabilize transgene expression by introducing a human codon-optimized dihydrofolate reductase (*DHFR*) gene into the viral coding cassette (Figure 1a). Since *DHFR* constitutes an essential gene of the purine metabolism, we reasoned that H9M cells with inactivated *Dhfr* (H9M^−/−^) could be cultivated in hypoxanthine and thymidine (HT) supplemented media prior to transduction, and that cells would become addicted to vector-mediated *DHFR* expression after HT withdrawal, resulting in stable color code expression.

### 3.2. Stable FGB-D Vector Expression in H9M^−/−^ Cells

We opted to evaluate the expression stability and the potential addiction to *DHFR* of selected 48xFGB constructs encoding for all six fluorescent marker combinations and the complete CAAR (HA^+^cMyc^+^Thy1.1^+^; 1BC, 5BC, 9BC, 13BC, 17BC and 21BC = 6xFGB(-D)) with (FGB-D) and without (FGB) *DHFR* coexpression cassette in H9M wild type cells and H9M^−/−^ cells. The latter were generated by nucleofection of ribonucleoprotein particles (RNP) of Cas9 complexed with a hybridized CRISPR RNA (crRNA) and a trans-activating crRNA (tracrRNA) (crRNA:tracrRNA) against the murine *Dhfr* gene and subsequent expansion in HT medium (Figure 1c). For vector tests, we chose a colony-forming cell assay (CFA)-derived clonal knockout line with two recombined *Dhfr* alleles (detected by TIDE) [21], resulting in graded DHFR expression intensities in flow cytometric analysis with fluorescein-labeled methotrexate (F-MTX; Figure S1a–d). While in H9M cells the 6xFGB and 6xFGB-D vector series both lost fluorescent marker expression, it persisted in 6xFGB-D transduced H9M^−/−^ cells in the presence and absence of HT supplement for at least 28 days (Figure 1d–h).

Next, we tested the effect of HT withdrawal on the enrichment of 6xFGB-D transduced polyclonal H9M^−/−^ cells. Here, the initial gene marking rate of 6xFGB-D vectors mildly increased, even in the presence of HT supplementation, while HT withdrawal led to a stronger enrichment (Figure 1i–k), which demonstrates the feasibility of our vector-mediated *DHFR* rescue approach. Likewise, the tracking of *Dhfr* editing rates in nucleofected cultures showed the growth advantage of non-genome edited cells in the presence and absence of HT-supplement (Figure S1e–g), which demands for color-coding of pure *Dhfr* knockout cultures, and suggests the incomplete rescue of *Dhfr* deficiency by the HT salvage pathway.

**Figure 2:**
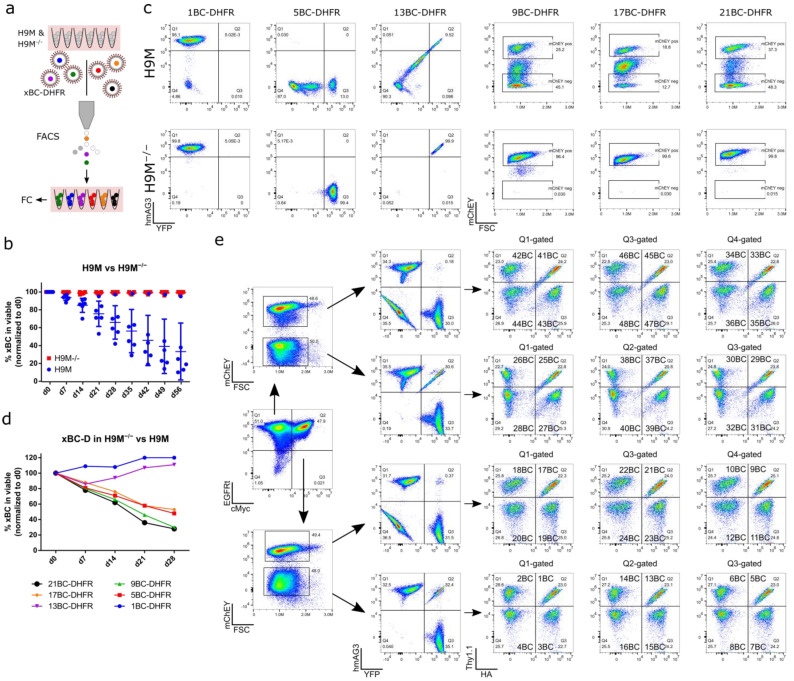
48xFGB-D vectors allow for deconvolution of transduced H9M^−/−^ cells. (**a**) Experimental design. H9M and H9M^−/−^ cells were transduced with 6xFGB-D vectors and subsequently sorted for pure color-coded populations. Color code expression was tracked over time by flow cytometry (FC). (**b**) Gene marking of 6xFGB-D vector transduced H9M and H9M^−/−^ cells over time. Six data points per condition; mean ± SD. (**c**) Exemplary flow cytometry plots of 6xFGB-D vector transduced H9M and H9M^−/−^ cells. (**d**) 6xFGB-D transduced H9M^−/−^ cells were sorted to purity and subsequently mixed with H9M cells. The content of color-coded cells at the beginning of the six individual assays was set to 100%. (**e**) Gating strategy for the deconvolution of 48 color-coded populations in 48xFGB-D transduced H9M^−/−^ cells. Cells were analyzed for fluorescent marker expression, as well as for CAAR surface marker tags (EGFRt, HA, cMyc, and dThy1.1).

We next enriched for color-coded populations to assess if vector silencing or a vector-induced competitive disadvantage caused the loss of color-coded cells (Figure 2a). In line with our previous observation (Figure 1f–h), 6xFGB-D vectors showed stable gene marking in sorting-enriched polyclonal H9M^−/−^ parental cells (H9M sample Rep2 10 µL in Figure S1h), but not in H9M cells, over 56 days of observation (Figure 2b). Interestingly, in H9M cells, the percentage of bright expressing cells slowly decreased in five out of the six samples, due to the production of an intermediate bright and subsequently negative cell population (Figure 2b,c). The exception was 1BC-D transduced H9M cells, which stably maintained high expression over time. These cells harbored FGB vector integrations in Bub1b (intron 12) and Rap1a (intron 3), which contributed with 81% and 19% to the overall clonal repertoire, suggestive of the parallel expansion of two clones in this sample. Similarly to the data depicted in Figure 1e, it appears that insertional mutagenesis might occasionally counteract the expected loss of FGB-D-marked *Dhfr*^wt^ cells.

Although virtually each of the 48 FGB-D vectors showed a stabilizing effect on transgene expression in H9M^−/−^ cells, the coexpression of *DHFR* did not lead to a general growth advantage. This could be seen in mixing experiments, where H9M cells caused a gradual loss of FGB-D vector-transduced H9M^−/−^ cells in four of six cultures (Figure 2d). Together, these data demonstrate that our *DHFR*-dependent metabolic addiction approach constitutes an efficient strategy for the maintenance of pure color-coded cell populations, without imposing a general growth advantage over non-edited cells.

### 3.3. FGB-D Vectors Produce Highly Specific Color Codes in Flow Cytometric Detection

We next tested for the possibility to detect and demultiplex all 48 FGB-D vector-transduced samples. Therefore, each vector was individually transduced into H9M^−/−^ bulk cultures, and gene marking was subsequently tracked based on fluorescent protein expression in individual samples or in combination with antibody-mediated detection of the different CAAR surface marker repertoires in mixed samples. Individual samples showed stabilized gene marking rates over time, with similar fluctuations between samples grouped according to fluorescent protein and CAAR configuration, respectively (Figure S2a–c). In contrast and expectedly, gene marking was lost for all constructs over time when monitored in 32D cells with endogenous *Dhfr* expression (Figure S2d–f). Most importantly, mixed H9M^−/−^ samples enabled the unequivocal flow cytometric detection of all 48 FGB-D color codes (Figure 2e).

Together, these data show that our 48xFGB-D vector series allows for the flow cytometric detection of eight CAAR configurations in combination with six fluorescent marker combinations, resulting in 48 highly specific color-coded AML populations putatively suitable for clonal tracking.

### 3.4. FGB-D Enables the Tracking and Identification of Growth-Enhanced H9M Clones In Vitro

**Figure 3:**
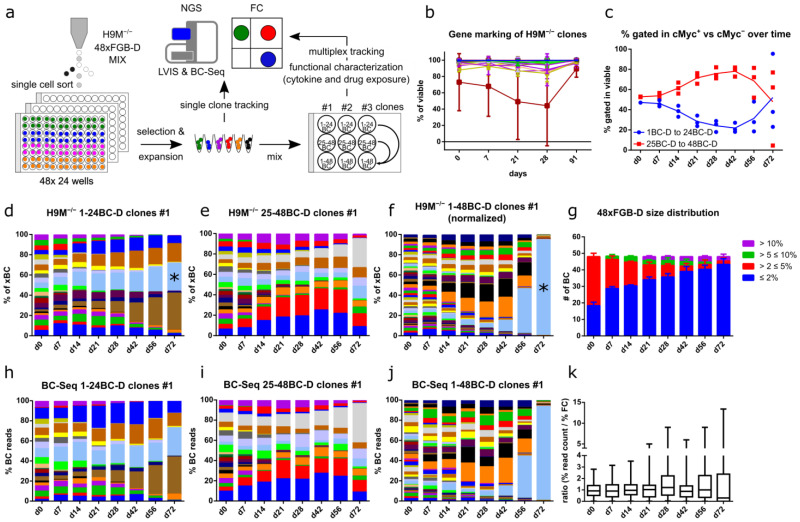
Color-coded clones show dynamic growth behavior and independent evolution of paired samples. (**a**) Experimental design. Transduced H9M^−/−^ cells were single-cell sorted for the derivation of clonal color-coded lines. These cells were subjected to lentiviral integration site analysis (LVIS), flow cytometric (FC) tracking and sequencing of the barcode repertoire (BC-Seq). (**b**) Gene marking rate of 144 H9M^−/−^ FGB-D clones over time. Clones 13BC-D (#2), 20BC-D (#1) and 45BC-D (#2) were excluded from the day 91 analysis, due to the presence of contaminating cells. (**c**) Contribution of cMyc^+^ (1-24BC-D) and cMyc^−^ (25-48BC-D) cells to the 48xFGB-D cell mix initiated with clones #1, #2 and #3. (**d**–**f**) Flow cytometric tracking of color-coded clones #1 cultures initiated with vectors (**d**) 1-24BC-D, (**e**) 25-48BC-D, and (**f**) 1-48BC-D. (**g**) Distribution of barcoded-population sizes over time for the cell mix from (**f**). (**h**–**j**) Determination of color-coded cell content in H9M^−/−^ cultures from (**d**–**f**) by BC-Seq. (**k**) Ratio of population sizes of H9M^−/−^ FGB-D transduced cells determined by (**h**–**j**) sequencing and by (**d**–**f**) flow cytometry. *, 17BC-D clone shared between the 1-24BC-D group and the 1-48BC-D group. Data depicted in (**d**–**f**) are representative for one of three experiments with different clones.

To track clonal growth behavior of AML in vitro, we performed single-cell sorting of 48 FGB-D labeled H9M^−/−^ mass cultures into 96 well plates. We expanded a minimum of three separate clones per color code, of which 144 (48x clones #1, #2 and #3 per BC-D) were further characterized (Figure 3a). Integration site analysis of these pooled clones recovered 92 unique FGB vector integrations. Surprisingly, in-gene integrations, as well as proto-oncogenic insertions, were highly overrepresented in comparison to a mixed random control, which is suggestive of a growth-promoting influence of the integration site (Figure S3a,b). Gene marking based on gating on the brightest fluorescent population was remarkably stable in 142 out of 144 analyzed clones for up to 91 days of observation (Figure 3b and Figure S4a). The two clones with reduced gene marking contained a second (dim) fluorescent population that may have been the result of variegation and was thus not included in the main gate (Figure S4b). Similar to polyclonal cultures, FGB-D labeled AML clones thus demonstrate highly stable marker expression, suitable for longitudinal tracking applications.

We next investigated the competitive growth behavior of these stable H9M clones in multiplex assays by generating nine different cell mixes consisting of vectors 1BC-D to 24BC-D (all cMyc^+^), 25BC-D to 48BC-D (all cMyc^−^), as well as 1BC-D to 48BC-D for clones #1, #2 and #3, respectively. While all 24xFGB-D mixes were prepared individually, the 48xFGB-D mixes were generated through pooling aliquots from the two 24xFGB-D mixes with 1BC-D to 24BC-D and 25BC-D to 48BC-D (Figure 3a). This way, we could directly compare the growth properties of the same H9M clones between the two 24xFGB-D and the combined 48xFGB-D samples. Tracking of these cell mixes by flow cytometry detected a higher growth potential of clones derived from the 25BC-D to 48BC-D parental mix within the 48xFGB-D samples at early time points (day 0–day 42) and the expansion of cMyc^+^ cells in one of the three 48xFGB-D mixes afterwards (Figure 3c). As exemplified for #1 clones, the 24xFGB-D mixes underwent a gradual reduction in clonal complexity, which culminated in the emergence of three and four dominant clones (>10% contribution) within the cMyc^+^ and cMyc^−^ samples, respectively (Figure 3d,e). Interestingly, in the corresponding 48xFGB-D sample, color codes derived from the cMyc^−^ sample initially expanded but were finally outcompeted by a single cMyc^+^ color code (17BC-D; labeled by asterisk) (Figure 3f). Strikingly, the 17BC-D clone stably persisted in its parental 24xFGB-D mix, indicating that it may have acquired a spontaneous growth-promoting mutation in the 48xFGB-D culture. As seen for the 24xFGB-D mixes, the 48xFGB-D mixes also demonstrated an increase of small color-coded (≤2%) populations, accompanied by the emergence of a small number of dominant color-coded clones (>10%) over time (Figure 3g). This observation reflects the dynamic growth characteristics of the clones, which follows at least four defined clonal patterns: 1. longitudinal extinction; 2. continuous expansion; 3. transient expansion and collapse; and 4. delayed expansion (Figure S4c). Notably, a similar dynamic distribution of color codes was also found by longitudinal next-generation sequencing analyses of the vector-specific BC region in these samples, suggesting that our flow cytometric analysis reliably captures clonal H9M growth in complex cultures (Figure 3h–k).

### 3.5. Color-Coded H9M Clones Follow Pre-Determined Behavior in Short-Term Assays

**Figure 4:**
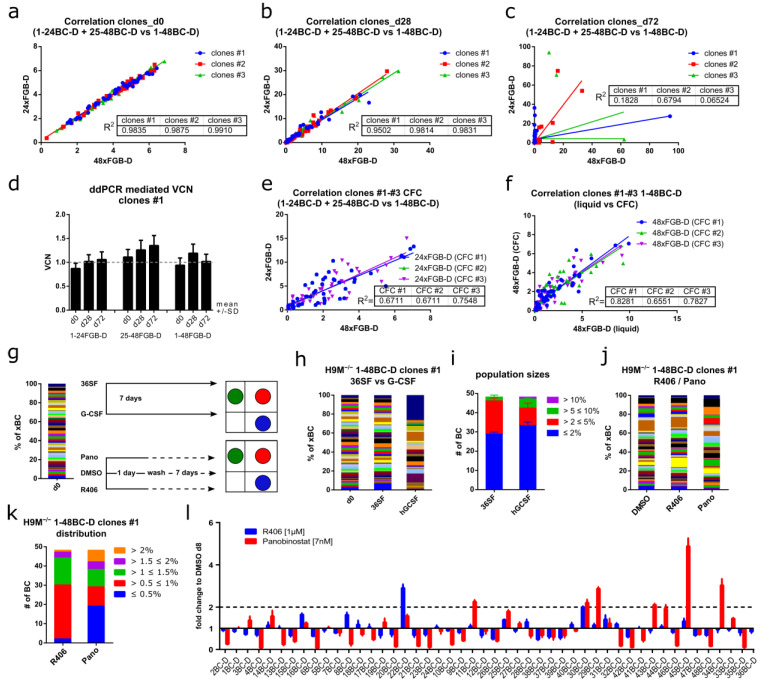
FGB-D H9M^−/−^ clones show differential growth behavior over time and in response to perturbagens. (**a**–**c**) Correlation of population sizes between the two 24xFGB-D (1-24BC-D and 25-48BC-D) samples and the corresponding 48xFGB-D sample for clones #1, #2 and #3 (**a**) at the start of the assay, (**b**) after 28 days and (**c**) after 72 days of tracking. (**d**) Determination of FGB-D vector copy numbers (VCN) in H9M^−/−^ clonal cell mixes over time by digital droplet PCR. (**e**) Correlation of clone sizes between the two 24xFGB-D (1-24BC-D and 25-48BC-D) samples and the corresponding 48xFGB-D sample for clones #1, #2 and #3 in CFA. (**f**) Correlation of clone sizes between the 48xFGB-D samples derived from clones #1, #2 and #3 in CFA and liquid culture. (**g**) Experimental design for the evaluation of cytokine-exposure and drug-challenge for clonal compilations of 48xFGB-D H9M^−/−^ cell mixes. (**h**,**i**) Comparison of FGB-D marker contributions after expansion of cell mixes in 36SF standard growth medium or in granulocyte colony stimulating factor (G-CSF)-supplemented base medium for 7 days. (**h**) Barcode distribution for one of three representative experiments. (**i**) Population sizes (mean ± SD) from three experiments with 48 data points each. (**j**–**l**) 48xFGB-D H9M^−/−^ cell mixes were exposed to the SYK inhibitor R406 or to the histone deacetylase inhibitor Panobinostat for 24 h, after which the cells were allowed to recover for 7 days. Flow cytometric analyses were used to determine (**j**) clonal expansion and collapse, (**k**) the size-distribution of barcoded clones (both mean values of triplicates), and (**l**) the fold-change between treated cultures and the DMSO control group measured in triplicates (mean ± SD).

We next wondered if stochastic or pre-determined cellular properties caused the dynamic clonal growth behavior in multiplex assays (Figure 3c–f). Therefore, we correlated the population sizes from the 24xFGB-D samples to their corresponding 48xFGB-D mix. In the beginning of the experiment (day 0), population sizes between the 24x and 48xFGB-D cell mixes were almost identical (R^2^ (clones #1) = 0.98, R^2^ (clones #2) = 0.99, and R^2^ (clones #3) = 0.99) (Figure 4a). This high degree of correlation (R^2^ (clones #1) = 0.95, R^2^ (clones #2) = 0.98, and R^2^ (clones #3) = 0.98) persisted 28 days later, although population sizes had already started to fluctuate (Figure 3d–f and Figure 4b). However, after 72 days, population sizes did not correlate anymore, presumably due to the spontaneous expansion of dedicated clones within the 24x and 48xFGB-D mixes (Figure 3d–f and Figure 4c). Notably, bulk cultures showed stable vector copy numbers over time, suggesting that a single FGB-D vector integration per cell suffices for rescuing the *Dhfr* knockout of the parental H9M cells (Figure 4d).

H9M cultures contain different subpopulations capable of maintaining liquid cultures and the formation of colonies in CFA, respectively [32]. The latter correlates with leukemic stem cell properties. When comparing color code distributions between the 24x and 48xFGB-D mixes in CFA, as well as between the CFA-derived 48xFGB-D mixes and liquid cultures, we observed poor correlations (Figure 4e,f). This supports the notion that CFA impose a strong selection pressure on H9M clones that accelerates stochastic clonal expansion.

Together, long-term culture and CFA trigger unpredictable clonal expansion, caused by stochastic selection mechanisms [19,33], while short-term cultures reveal pre-determined and predictable growth properties.

### 3.6. FGB-D Labeled H9M^−/−^ Clones Differentially Respond to Cytokine Stimulation and Drug Challenge

The pre-determined growth properties in short-term assays destine FGB-D labeled H9M^−/−^ clones for comparative assessment of their responses to perturbagens (Figure 4g). Expansion of the 48xFGB-D cell mixes in either standard growth conditions (36SF) or in 50 ng/mL G-CSF for 7 days produced an increasing number of small (≤2%) and large (>5%) color codes in the treated samples, suggestive of a heterogeneous potential of clones to grow in response to G-CSF (Figure 4h,i). Additionally, we prepared a new 48xFGB-D mix from selected clones and subsequently treated this culture with the SYK inhibitor R406 and the histone deacetylase inhibitor Panobinostat for 24 h, after which the cells recovered for 7 days (Figure 4g). While the color code distribution in the control sample remained stable throughout the experiment, both treatment groups reported the selective expansion and collapse of dedicated clones (Figure 4j–l). Thus, 48xFGB-D multiplex assays allow for the identification of perturbagen-resistant clones as an efficient approach to mimic clonal selection.

### 3.7. Growth Characteristics Are Stably Maintained in Purified Clones

**Figure 5:**
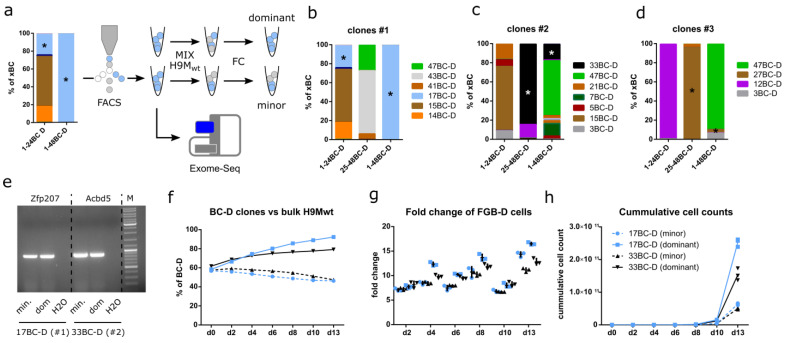
De novo acquired growth properties are stably maintained in dominant clones. (**a**) Experimental design. Minor and dominant clones were sorting purified from paired 24xFGB-D and 48xFGB-D H9M^−/−^ cultures and subjected to competition assays, growth assays and exome sequencing. (**b**–**d**) Distribution of color-coded H9M^−/−^ clones after 137 days of tracking derived from (**b**) clones #1, (**c**) clones #2, and (**d**) clones #3 samples. Sorting purified clones are marked by *. (**e**) Locus specific PCR for the amplification of the vector–genome boundary, demonstrating clonal origins of minor (min.) and dominant (dom.) sorting purified samples from clones #1 and #2. (**f**) In vitro competition experiment of sorting purified 17BC-D and 33BC-D minor and dominant clones, each individually competed against H9M wild type cells (3 data points, each; mean ± SD). (**g**,**h**) Cell growth depicted as (**g**) fold-change (mean ± SD) and (**h**) cumulative cell count of sorting purified color-coded clones.

Differences in clonal growth rate and the response to external stimuli can be caused by pre-determined (e.g., cell of origin) and acquired (e.g., mutations) properties [34,35]. Delineating these effects remains challenging, due to the time requirements for clonal evolution to proceed and a lack of suitable reference samples [36]. Aided by our paired 24xFGB-D and 48xFGB-D samples, we attempted to overcome both limitations by selecting paired clones from all three lines that persisted in one cell mix and dominated the other (Figure 5a). These criteria were fulfilled by clones 17BC-D (clones #1), 33BC-D (clones #2), and 27BC-D (clones #3) (Figure 5b–d), which harbor integrations in *Zfp207* (intron 13), *Acbd5* (intron 3), and *Gm4890* (intron 2), respectively. While the 27BC-D minor population failed to be enriched, due to its small size, FACS of minor and dominant 17BC-D and 33BC-D clones followed by the amplification and sequencing of their vector–genome boundary succeeded (Figure 5e). The capture of identical barcode sequences and integration sites in paired samples verified their common clonal origin (Figure S5a,b). The 17BC-D and 33BC-D clones were subsequently competed against non-transduced H9M cells. As in complex mixtures, dominant clones outcompeted their competitors, while the contribution of minor clones slowly declined over time (Figure 5f). Accordingly, dominant clones also proliferated faster and produced higher cumulative cell numbers (Figure 5g,h). These observations support the hypothesis that dominant clones acquired stably inherited growth-promoting alterations.

### 3.8. Exome Sequencing Identifies Unique Differences between Paired Clones

We next performed exome sequencing on the paired clonal samples 17BC-D and 33BC-D to unearth potential genetic differences that may explain the spontaneous expansion of these dominant clones. Exome sequencing returned identical recombination events of the *Dhfr* locus within the paired samples, further confirming their clonal origin (Figure S5c). Furthermore, exome sequencing recovered 133 and 175 somatic SNPs between the 17BC-D and 33BC-D paired samples, respectively. Of those, 30 and 33 affected the coding sequence of annotated genes and were either synonymous (10 (17BC-D) and 6 (33BC-D)), non-synonymous (19 (17BC-D) and 26 (33BC-D)) or stop-gain (1 each). This was accompanied by InDels in 162 and 186 loci (3 and 1 within coding sequences), and copy number variations in 24 and 18 loci of the 17BC D and 33BC-D samples, respectively (Table S2). However, none of the genetic aberrations establishes a clear link to growth promotion or is likely to be associated with known oncogenic functions. Our data thus suggest that non-genetic mechanisms may contribute to the observed stably inherited heterogeneous clonal behavior over time.

### 3.9. In Vivo Leukemogenesis Combines Stochastic and Pre-Determined Clonal Selection Mechanisms

**Figure 6:**
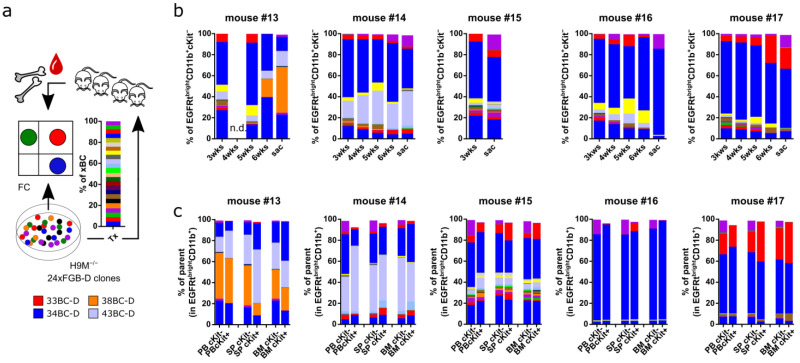
In vivo restriction of color-coded clonal complexity. (**a**) Experimental design. A 24xFGB-D (25BC-D—48BC-D) H9M^−/−^ clonal cell mix was generated through cell pooling. After determination of the color-coded clone sizes in the mix by flow cytometry, aliquots of the mixture were injected into five lethally irradiated mice for longitudinal tracking. (**b**) Peripheral blood (PB) analysis of color-coded cell content in the graft-derived myeloid (CD45.1^+^EGFRt^bright^CD11b^+^) cell population over time. (**c**) Distribution of color-coded cell content in the graft-derived myeloid (CD45.1^+^EGFRt^bright^CD11b^+^) cell population in PB, spleen (SP), and bone marrow (BM) samples of endpoint mice (except for mouse #15) according to the expression or absence of cKit. Mouse #15 was already sacrificed 4 weeks post transplantation to obtain reference cell material.

Given the stochastic expansion of clones in long-term cultures, we next wondered if the same principles would also apply to the in vivo selection of clones. To this end, we performed a pilot experiment and pooled clones from samples 25BC-D to 48BC-D for transplantation into five recipient mice (Figure 6a). The first peripheral blood analysis 3 weeks after transplantation already revealed the presence of the same markedly expanded clone (34BC-D) in all recipients (Figure 6b). Interestingly, this clone reached dominance in two (mouse #16 and mouse #17) out of four endpoint samples, and shared clonal dominance with clone 43BC-D in mouse #14. In contrast, mouse #13 presented with a dominant 38BC-D clone. Notably, the fifth mouse (#15) was already sacrificed 4 weeks after transplantation to compare the color code distribution between the peripheral blood, bone marrow, and spleen at a pre-malignant state (Figure 6b,c). In addition to the presence of multiple minor clones, this revealed a high similarity between the CD11b^+^cKit^−^ and CD11b^+^cKit^+^ populations representative for the blast and LSC compartments, respectively, for each organ, as well as between the different organs. A similar correlation between the color code distributions also appeared in endpoint samples and suggests that engraftment-compatible clones are equally capable of populating the bone marrow, as well as extramedullary sites.

### 3.10. Distinct Gene Expression Programs Underlie Clonal Expansion and Maintenance

**Figure 7:**
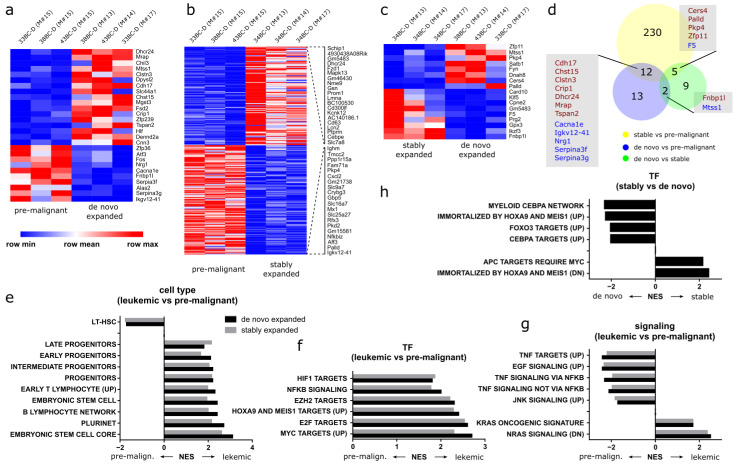
Gene expression analysis reveals longitudinal changes associated with clonal expansion. Transcriptional analyses were performed with cKit^+^CD11b^dim^ color code-enriched splenocytes. (**a**) Differential expression analysis between pre-malignant clones (33BC-D, 38BC-D and 43BC-D) from mouse #15 and the corresponding samples after de novo expansion in mice #13, 14 and 17. (**b**) Differential expression analysis between pre-malignant clones (38BC-D, 43BC-D and 33BC-D) from mouse #15 and the stably expanded clone 34BC-D from mouse #13, 14 and 17. (**c**) Differential expression analysis between the stably expanded clone 34BC-D from mouse #13, 14 and 17, and de novo expanded clones from the same mice. (**d**) Venn-diagram of genes overlapping in the different groups of differential gene expression analysis. Red genes are overexpressed, blue genes are downregulated. (**e**–**h**) Gene set enrichment analysis (GSEA) of differentially expressed genes between (**e**–**g**) pre-malignant samples and de novo and stably expanded clones, respectively. (**h**) GSEA of differentially expressed genes between stably expanded and de novo expanded clones. NES, normalized enrichment score. All NES with an FDR q-val ≤ 0.05.

To analyze the transcriptional foundation for clonal expansion, we performed RNA-Seq on sorting-purified minor clones (33BC-D, 38BC-D and 43BC-D) from a pre-malignant mouse (#15; sacrificed 4 weeks post transplantation), as well as from the corresponding de novo expanded clones of endpoint mice (Figure 6c). Additionally, the stably expanded clone 34BC-D was also sequenced from the same mice. Differential gene expression analysis identified a total of 27 2-fold up- and down-regulated genes, with an adjusted *p*-value (*p*-adj) < 0.05 between pre-malignant and de-novo expanded clones (Figure 7a). Expectedly, transcriptional differences were higher between pre-malignant clones and the stably expanded clone (247 2-fold up- and down-regulated genes with *p*-adj < 0.05) (Figure 7b), while only 17 differentially expressed genes remained between the de novo expanded clones and the stably expanded clone 34BC-D from three mice (Figure 7c). This suggests that only a relatively small number of differentially regulated genes determines clonal fate decisions and expansion (Figure 7d), and underlines the advantage of working with purified clones for differential analyses.

To better investigate the transcriptional differences on a global scale, we next performed gene set enrichment analyses (GSEA). The comparison of leukemic cells against pre-leukemic cells associated the former with various progenitor populations, as well as pluripotency (Figure 7e). Furthermore, leukemic populations enriched for transcription factors HIF1, E2F and MYC, as well as for EZH2-mediated epigenetic regulation and RAS signaling, all of which represent common oncogenic players (Figure 7f,g) [37]. In contrast, pre-leukemic cells display features of hematopoietic stem cells, along with tumor necrosis factor (TNF) and c Jun N-terminal kinase (JNK) signaling (Figure 7e,g). Strikingly, gene sets compared between the two (de novo and stably expanded) leukemic clonal populations uncovered their association with different *Hoxa9* and *Meis1-*dependent gene sets. In comparison to transformation by H9M, de novo transformed cells enriched for genes upregulated in *Hoxa9* transformed progenitors (Immortalized by Hoxa9 and Meis1 (UP)), while stably expanded clones enriched for genes downregulated in *Hoxa9* transformed progenitors (Immortalized by Hoxa9 and Meis1 (DN)) (Figure 7h). The combination of a *Hoxa9*-dominated transcriptional profile along with the maintenance of CEBPα-dependent myeloid differentiation stimuli thus appears instrumental for delayed clonal expansion (Figure 7h).

## 4. Discussion

We here report the real-time flow cytometric tracking of clonal competition and the monitoring of clonal evolution in AML based on a complex fluorescent genetic barcoding vector system. The cFGB strategy thus closes the gap between single-clone assays, single-cell sequencing and deep-sequencing of bulk populations and readily helped identify longitudinal transcriptional changes underlying clonal maintenance and expansion.

Key to the real-time tracking of leukemic clones was the development of the cFGB vector platform. For this purpose, we combined our fluorescent marker cassettes with eight newly generated CAARs as multifunctional detection molecules (Figure 1). Notably, OBC represents an alternative complex color-coding system for clonal tracking [13]. However, OBC-derived color codes typically require multiple vector integrations per cell, which makes them at risk for misinterpretation of cellular identity due to vector silencing or variegation. Moreover, OBC vectors do not contain DNA barcodes that would allow for sequencing-based quality control. We tackled these limitations by creating a library following the “one color code per vector” concept that resists silencing through *DHFR* addiction, links DNA barcode and fluorescence identities, allows for pre-gating on vector-bearing cells through the shared EGFRt marker, and enables the deconvolution of clonal repertoires by hierarchical gating (Figure 1, Figure 2e and Figure 3d–k). Despite these differences, OBC and FGB both rely on the expression of different marker proteins and thus cannot achieve the same complexity as DNA barcode vector libraries [38]. Regardless, previous barcoding studies in the mouse MLL-AF9 AML model have shown that ~50 clones per recipient accounted for the 95th percentile of bulk barcode sequences [19]. Therefore, 24–48 color codes might be sufficient to recapitulate the fate of a representative distribution of disease-causing cells. Because this information is not accessible for most target cell systems, FGB approaches should generally be used when tracking of a limited number of clones is to be achieved with real-time resolution and with the perspective to reisolate cells of interest.

Our in vitro clonal multiplexing studies allowed for the tracking of the majority of color codes for several weeks (Figure 3d–f,h–j), and reported the expansion of selected clones between paired cultures. Interestingly, the dominant clones, in comparison to their persisting counterparts, maintained these enhanced growth properties, even after enrichment by FACS and subsequent competition against H9M^wt^ cells for 13 days (Figure 5). Although we did not generate long-term data on the competitiveness of these growth-enhanced clones, our experiments support a concept, where long-term culture selects for highly competitive clones that maintain this behavior, even after extraction from the mixed cultures. We thus refer to these growth characteristics as “stably maintained”. Further studies will reveal whether these cells die out due to exhaustion, are overgrown by even more aggressive mutants, and show a unique response to drugs.

In comparison to the starting cell mix, we observed a highly divergent distribution of color codes as early as 3 weeks post-transplantation, which evolved over time into a unique pattern in individual mice (Figure 6b,c). Thus, clonal behavior in vivo likely depends on pre-determined and stochastic features, similar to those observed in vitro (Figure 3 and Figure 4), although hallmarks such as homing, engraftment and possibly immune escape appear to be increasingly important.

Although our pilot study has only been conducted with a limited number of mice, the in vivo reduction of color codes also occurred in larger H9M transplantation experiments utilizing lower complexity FGB systems, as well as in DNA-barcoding of MLL-AF9 AML [15,19]. Thus, a reduction in clonal complexity represents a common feature of AML transplantation assays, which allows delineation of transcriptional peculiarities of highly competitive vs. non-expanding cells.

As we were most interested in understanding the mechanisms that underlie clonal expansion, we generated differential gene expression profiles between the same clones from an early (pre-malignant) time point and the leukemic (de novo expanded) endpoint. In doing so, transcriptional noise from the different clonal origins could be reduced, as made apparent by the relatively low number of differentially expressed genes between paired pre-malignant and de novo expanded clones in comparison to stably expanded clones (Figure 7a,b). Regardless, stably expanded clones and de novo expanded clones only harbored 17 differentially expressed genes. This analysis suggests that the early expansion of the stably expanded clone 34BC-D in all animals was presumably driven by the overexpression of glutathione peroxidase 3 (*Gpx3*), which emerged as the most significantly (*p* adj 1.62^−46^) upregulated (13.2x) gene between the two leukemic populations (Figure 7c). Notably, *Gpx3* upregulation was previously associated with increased self-renewal potential of H9M BM lines, due to promoter hypomethylation as well as with poor prognosis in human AML [39]. However, since we did not correlate the in vitro *Gpx3* expression of our clones with their in vivo growth behavior, further work needs to investigate *Gpx3′s* potential as a predictive marker for the selection of aggressive H9M clones from in vitro cultures.

Moreover, we discovered an enrichment of tumor necrosis factor (TNF)-driven signaling in pre-malignant cells. In both leukemic (stably expanded and de novo) samples, however, RAS signaling, as well as NF-κB and cMyc expression signatures, became dominant. This suggests a longitudinal change in signaling dependencies, which might offer a therapeutic approach that is specifically targeted at the clone with the highest predicted risk for malignant conversion and expansion.

Mechanistically, the autocrine secretion of pro-inflammatory TNF triggers a NF-κB-dependent positive feedback loop in MLL-rearranged (MLLr) AML, which helps to establish the MLL expression signature [40]. Moreover, TNF activates JNK, which provides additional proliferation and survival signals [41]. Together, the TNF–NF-κB/JNK pathway supports the expansion of clonogenic LSC. While, in our model, TNF appears especially important for the establishment of disease (e.g.**,** in pre-malignant cells), combined RAS-, NF-κB- and Myc-signatures dominate the leukemic populations. This is in line with the observation that TNF superfamily members act especially during the initiation phase of leukemia [42], while the stabilization of NF-κB through a RAS/PI3K/AKT-dependent pathway may become dominant at later stages [43]. This would be consistent with the frequent observation of RAS pathway mutations in AML patients and their importance in driving a positive feedback loop mediated by the NF-κB pathway [44,45,46,47,48].

Our clonal tracking approach thus recapitulated transcriptional-dependencies of MLLr on NF-κB, and uncovered additional longitudinal changes of NF-κB activation through TNF and RAS in the early and late stage of the disease, respectively.

Given that we did not perform mutational analyses of H9M cells after transplantation, we cannot rule out that the observed shift in signaling dependencies occurred through the acquisition of genetic aberrations. However, it has long been postulated that the H9M system does not rely on the acquisition of additional genetic hits, due to its aggressive nature and short latency to reach end-stage disease in mice. Moreover, recently non-genetic transcriptional alterations have been linked with clonal evolution, suggesting that transcriptional analyses might be more powerful in characterizing leukemogenesis than mutational profiling [33]. This would explain the lack of obvious growth-promoting alterations in our exome sequencing data of paired clones (Figure 5).

Despite the unclear implications of clonal evolution for our experiments, leukemia is generally composed of multiple competing subclones, which form a cellular reservoir for relapse [49]. Recent publications showed that minor relapse-driving subclones with increased drug resistance can already be present in diagnostic samples, but their awakening in response to therapy remains enigmatic [50,51]. Our color-coded leukemia model thus provides an interesting approach to test the consequences of targeted clone depletion on the reactivation of dormant subclones and the underlying “switchboard” signals with single cell resolution that may have therapeutic potential [52,53]. Therefore, future color-coding experiments should be adapted to recently developed scRNA-Seq applications, allowing for the simultaneous assessment of clonal identity (through BC sequencing), mutational profiling (e.g., MutaSeq), phenotypic characterization (e.g., CiteSeq), and transcriptome analysis, to enable a detailed view of interclonal and intraclonal heterogeneity over time and the underlying mechanisms regulating clonal compilations [12,19,54].

This range of applications highlights the ability of our cFGB approach to bridge the gap between low-throughput clonal transplantation assays and high-throughput sequencing studies for clonal fate tracking in malignant hematopoiesis, which will also be applicable to other organ systems with stem cell-mediated regeneration.

## Data Availability

The datasets associated with the current study are available from the corresponding author upon reasonable request. Raw and processed RNA sequencing data can be accessed via the Gene Expression Omnibus under GEO: GSE198371.

## Supplementary Materials

The following supporting information can be downloaded at: https://www.mdpi.com/article/10.3390/cells11244045/s1, Figure S1: Establishment of H9M^−/−^ cells; Figure S2: FGB-D vectors provide stable expression over time in H9M^−/−^ cells and yield specific signals in mixed cultures; Figure S3: Integration site preferences in color-coded H9M^−/−^ clones; Figure S4: Exemplified flow cytometric analysis of selected color-coded clones; Figure S5: Identity check of paired samples from competitive growth experiments; Table S1: BC sequences; Table S2: Exome seq; Table S3: Indexing primers.
