# Supplementary material for: Real-Time Characterization of Clonal Fate Decisions in Complex Leukemia Samples by Fluorescent Genetic Barcoding
Source: Cells. 2022 Dec 14;11(24):4045. doi: 10.3390/cells11244045 (PMC9776743; doi:10.3390/cells11244045)
Supplement: Supplementary file 1 [file cells-11-04045-s001.zip › Figure S1.pdf]

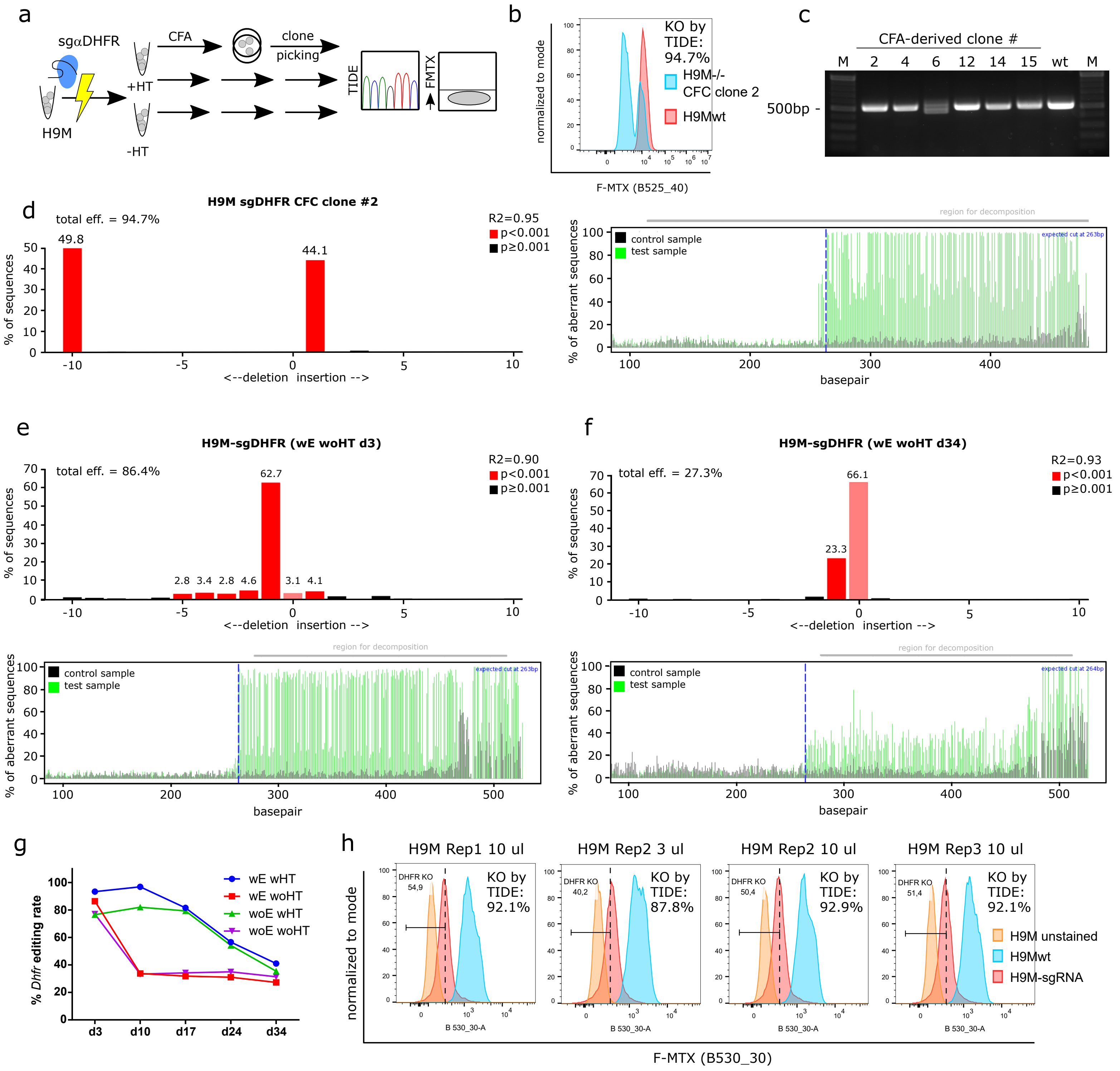

Figure S1: Establishment of H9M$^{-/-}$ cells. (a) Experimental design. H9M cells were nucleofected with Cas9 RNPs against the *Dhfr* locus. Cells were subsequently either put into colony-forming cell assays (CFAs) in the presence of HT to select for self-renewing clones or cultivated as bulk cultures with and without HT supplementation. CFA-derived clones and bulk cultures were analyzed by locus-specific PCR and TIDE analysis for recombination efficiencies. Fluorescein-labeled methotrexate (F-MTX) was used for the identification of DHFR expressing cells in flow cytometric analyses. **(b)** Overlay histogram plot of F-MTX stained H9M control cells (wt) and H9M$^{-/-}$ cells derived from a single CFA-colony. Knockout (KO) frequency of the DHFR allele is indicated. **(c)** Representative agarose gel electrophoresis for the detection of *Dhfr* PCR products (555 bp) followed by direct sequencing and TIDE analysis. **(d)** TIDE analysis of the H9M$^{-/-}$ CFA-derived clone #2 from **(b)** & **(c)**. **(e-f)** Representative TIDE analyses performed **(e)** 3 days and **(f)** 34 days after nucleofection in the presence of a nucleofection enhancer and expansion in the absence of HT supplement. **(g)** H9M cells were nucelofected in the presence (wE) and absence (woE) of a nucleofection enhancer prior to cultivation in the presence (wHT) and absence (woHT) of HT supplementation. The graph shows the percentage of edited DHFR alleles over time as determined by TIDE analysis of PCR products after Sanger sequencing. **(h)** Flow cytometric analyses of four different H9M parental lines subjected to nucleofection of Cas9 RNPs against *Dhfr* and staining with F-MTX after 7 days of expansion in the presence of HT supplement. Gates for the enrichment of KO cells by FACS (dotted line) as well as KO frequencies as determined by TIDE on the day of sorting are indicated.
