# Supplementary material for: Real-Time Characterization of Clonal Fate Decisions in Complex Leukemia Samples by Fluorescent Genetic Barcoding
Source: Cells. 2022 Dec 14;11(24):4045. doi: 10.3390/cells11244045 (PMC9776743; doi:10.3390/cells11244045)
Supplement: Supplementary file 1 [file cells-11-04045-s001.zip › Figure S2.pdf]

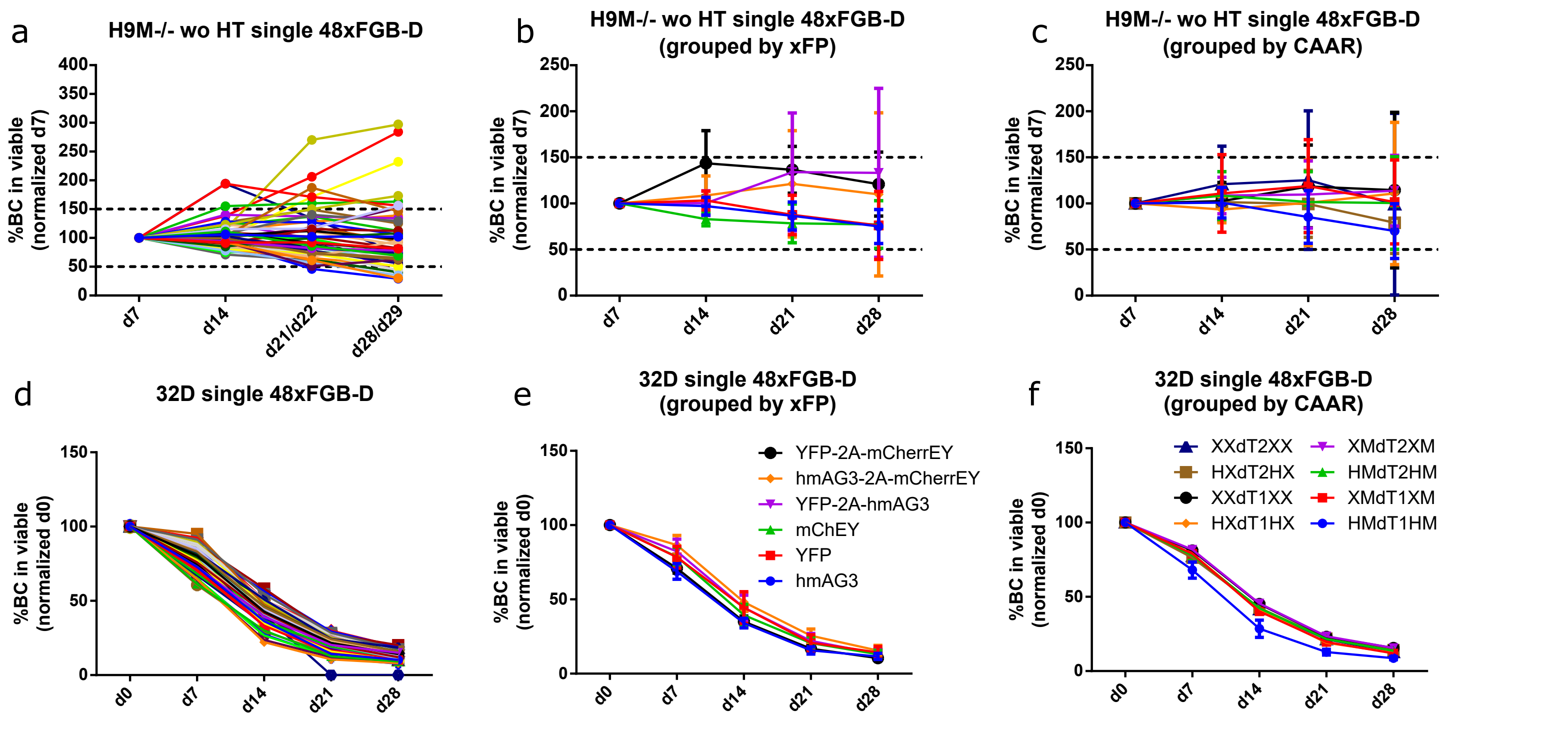

Figure S2: FGB-D vectors provide stable expression over time in H9M$^{-/-}$ cells and yield specific signals in mixed cultures. (a-c) H9M$^{-/-}$ cells were transduced with all 48 FGB-D vectors in individual wells for longitudinal tracking by fluorescent marker expression in the absence of HT-supplementation. All data were normalized to the gene marking rate in the beginning (d7) of the tracking experiment. **(a)** Normalized gene marking rates for all 48 FGB-D transduced samples. **(b)** Normalized gene marking rate of cells from **(a)** grouped according to fluorescent markers (n=6). **(c)** Normalized gene marking rate of cells from A grouped according to CAAR (n=8). **(d-f)** 32D cells were transduced with all 48 FGB-D vectors in individual wells for longitudinal tracking by fluorescent marker expression. All data were normalized to the gene marking rate in the beginning (d0) of the tracking experiment. **(d)** Normalized gene marking rates for all 48 FGB-D transduced samples. **(e)** Normalized gene marking rate of cells from **(a)** grouped according to fluorescent markers (n=6). **(f)** Normalized gene marking rate of cells from **(a)** grouped according to CAAR (n=8). Notably, in **(d-f)** sample 48BC-D (YFP-2A-mCherrEY-P2A-XXdT2XX) could not be measured at day 21 and 28.
