# Supplementary material for: Real-Time Characterization of Clonal Fate Decisions in Complex Leukemia Samples by Fluorescent Genetic Barcoding
Source: Cells. 2022 Dec 14;11(24):4045. doi: 10.3390/cells11244045 (PMC9776743; doi:10.3390/cells11244045)
Supplement: Supplementary file 1 [file cells-11-04045-s001.zip › Figure S3.pdf]

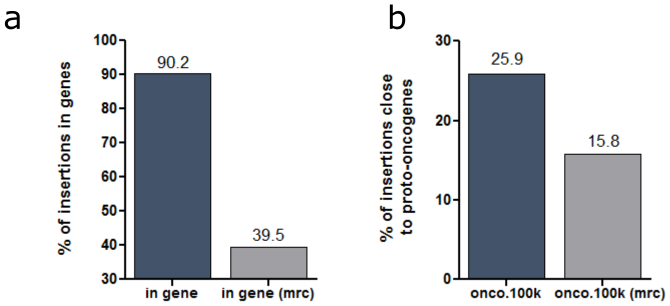

Figure S3: Integration site preferences in color-coded H9M$^{-/-}$ clones. (a) Distribution of in-gene insertions in H9M$^{-/-}$ clones (dark blue) and a mixed random control (grey). **(b)** Distribution of integrations in H9M$^{-/-}$ clones in the vicinity of proto-oncogenes (dark blue) and a mixed random control (grey).
