# Supplementary material for: Real-Time Characterization of Clonal Fate Decisions in Complex Leukemia Samples by Fluorescent Genetic Barcoding
Source: Cells. 2022 Dec 14;11(24):4045. doi: 10.3390/cells11244045 (PMC9776743; doi:10.3390/cells11244045)
Supplement: Supplementary file 1 [file cells-11-04045-s001.zip › Figure S4.pdf]

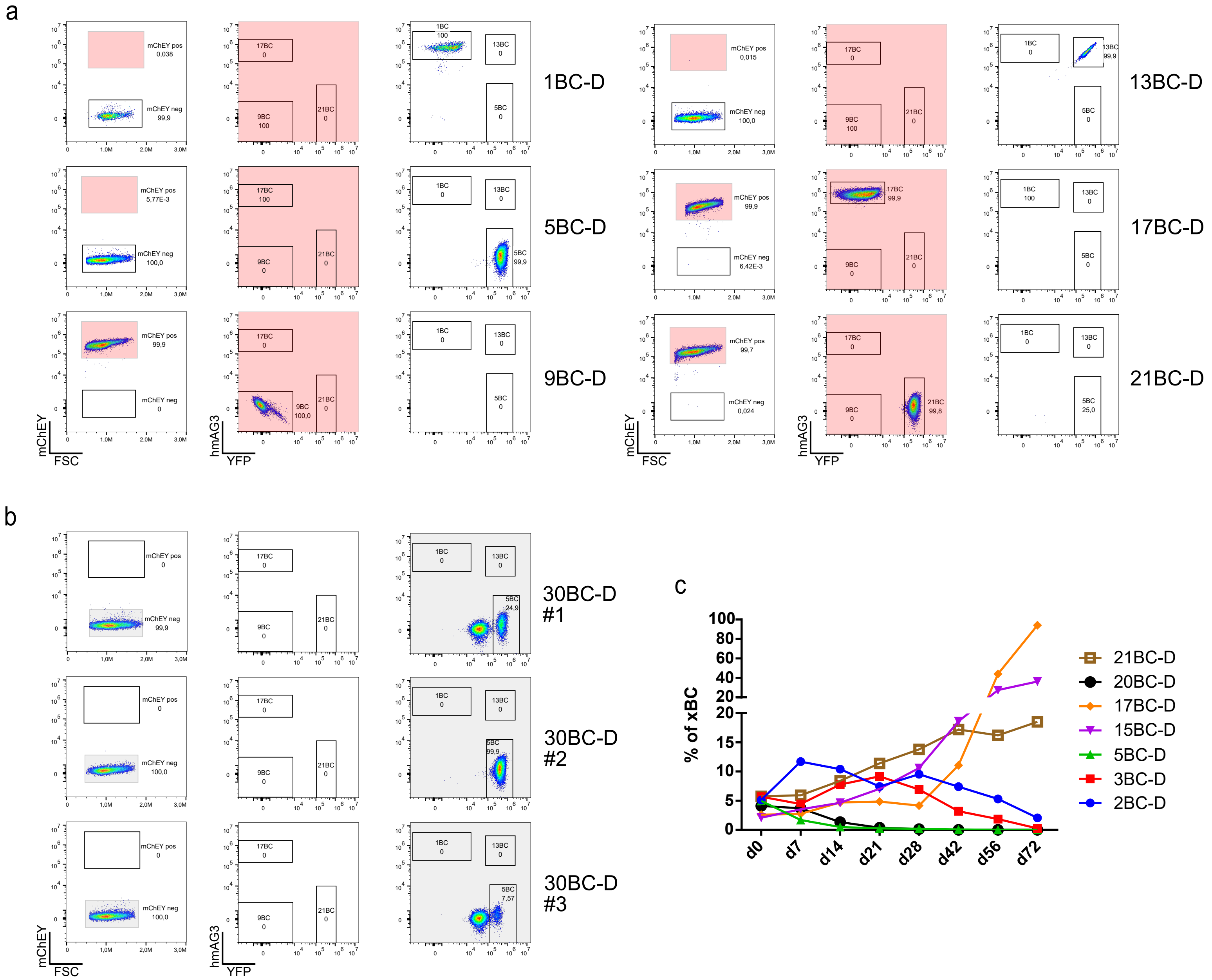

Figure S4: Exemplified flow cytometric analysis of selected color-coded clones. (a-b) FGB-D H9M$^{-/-}$-derived color-coded clones were subjected to flow cytometric analyses to determine signal specificities based on fluorescent marker expression. **(a)** Samples with single defined fluorescent populations. **(b)** Clones #1-3 of 30BC-D transduced H9M$^{-/-}$ samples with only a single fluorescent population in clone #2, while clones #1 and #3 showed rare signs of variegated vector expression. **(c)** Exemplified clonal growth pattern extracted from Figure 3d (transient expansion: 2BC-D and 3BC-D; extinction: 5BC-D and 20BC-D; longitudinal expansion: 15BC-D and 21-BC-D) and Figure 3f (delayed expansion: 17BC-D*).
