# Supplementary material for: Real-Time Characterization of Clonal Fate Decisions in Complex Leukemia Samples by Fluorescent Genetic Barcoding
Source: Cells. 2022 Dec 14;11(24):4045. doi: 10.3390/cells11244045 (PMC9776743; doi:10.3390/cells11244045)
Supplement: Supplementary file 1 [file cells-11-04045-s001.zip › Figure S5.pdf]

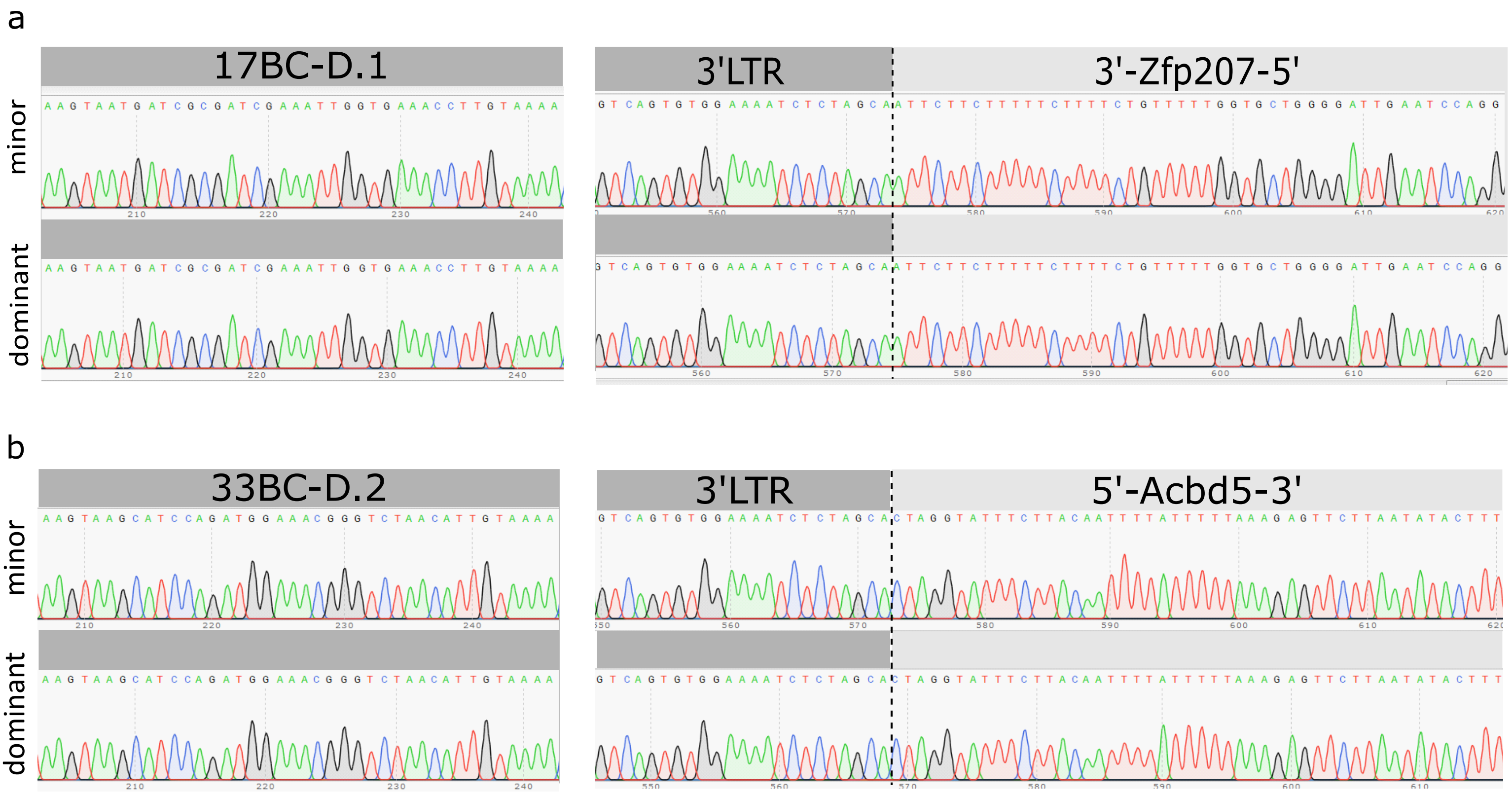

c

Chr	Start	End	Ref	Func. refGene	Gene. refGene	ExonicFunc. refGene	AAChange.refGene	VAF	
**17BC-D.1 (minor)**									
chr13	92355309	92355330	TGGCAAGAACGGAGACCTACCC	exonic	Dhfr	fs deletion	Dhfr:NM_010049:exon1:c.51_72del:p.Ile17fs	0.5	TTGGCAAGAACGGAGACCTACCC
chr13	92355328	92355332	CCCTG	exonic	Dhfr	fs deletion	Dhfr:NM_010049:exon1:c.70_74del:p.Pro24fs	0.5	ACCCTG
**17BC-D.1 (dominant)**									
chr13	92355309	92355330	TGGCAAGAACGGAGACCTACCC	exonic	Dhfr	fs deletion	Dhfr:NM_010049:exon1:c.51_72del:p.Ile17fs	0.5	TTGGCAAGAACGGAGACCTACCC
chr13	92355328	92355332	CCCTG	exonic	Dhfr	fs deletion	Dhfr:NM_010049:exon1:c.70_74del:p.Pro24fs	0.5	ACCCTG
**33BC-D.2 (minor)**									
chr13	92355311	92355338	GCAAGAACGGAGACCTACCCTGGCCTCC	exonic	Dhfr	fs deletion	Dhfr:NM_010049:exon1:c.53_80del:p.Gly18fs	1	GGCAAGAACGGAGACCTACCCTGGCCTCC
**33BC-D.2 (dominant)**									
chr13	92355311	92355338	GCAAGAACGGAGACCTACCCTGGCCTCC	exonic	Dhfr	fs deletion	Dhfr:NM_010049:exon1:c.53_80del:p.Gly18fs	1	GGCAAGAACGGAGACCTACCCTGGCCTCC

Figure S5: Identity check of paired samples from competitive growth experiments. (a-b) Sequencing analysis of minor and dominant clones **(a)** 17BC-D.1 and **(b)** 33BC-D.2, respectively. Paired minor and dominant samples contained the same barcode sequence as well as identical 3’LTR-genome boundaries. **(c)** Exome sequencing verified identical mutations in the DHFR loci for paired minor and dominant 17BC-D.1 and 33BC-D.2 clones, respectively. VAF, variant allele frequency.
